# Case report: Cerebrospinal fluid-derived circulating tumor DNA diagnoses and guides the treatment of a lung adenocarcinoma case with leptomeningeal metastasis

**DOI:** 10.3389/fonc.2022.944963

**Published:** 2022-11-28

**Authors:** Yujun Bai, Qingxi Yu, Ning Liu, Jingwen Liu, Di Wang, Xiaoli Liu, Shuanghu Yuan

**Affiliations:** ^1^ Department of Radiation Oncology, Tai’an Central Hospital (Tai’an Central Hospital Affiliated to Qingdao University, Taishan Medical Care Center), Tai’an, Shandong, China; ^2^ Department of Radiation Oncology, Shandong Cancer Hospital and Institute, Shandong First Medical University and Shandong Academy of Medical Sciences, Jinan, Shandong, China; ^3^ Department of Radiation Oncology, Shandong Cancer Hospital Affiliated to Shandong University, Jinan, Shandong, China; ^4^ Geneseeq Research Institute, Nanjing Geneseeq Technology Inc., Nanjing, Jiangsu, China; ^5^ Cheeloo College of Medicine, Shandong University, Jinan, Shandong, China; ^6^ Department of Radiation Oncology, The Affiliated Cancer Hospital of Zhengzhou University, Henan Cancer Hospital, Zhengzhou, Henan, China

**Keywords:** NSCLC, ctDNA, leptomeningeal metastasis (LM), cerebrospinal fluid (CSF), case report

## Abstract

Leptomeningeal metastasis (LM) occurs in 3~5% of non-small cell lung cancer (NSCLC) patients. Diagnosis of patients with LM and disease monitoring remains challenging due to the low sensitivity and specificity of the commonly used approaches, such as cerebrospinal fluid (CSF) cytology and magnetic resonance imaging (MRI). Therefore, new approaches are necessary to improve the detection of LM. Recent studies have shown that circulating tumor DNA (ctDNA) in CSF can be used to detect and monitor LM, but whether it can serve as an early diagnostic biomarker prior to cytological and radiographic evidence of LM involvement requires further evaluation. Here we report a lung adenocarcinoma patient who had detectable oncogenic mutations in the CSF ctDNA prior to confirmation of LM by CSF cytology and MRI, highlighting the potential application of CSF ctDNA in early detection of LM.

## Introduction

Leptomeningeal metastasis (LM) can be found in 3~5% of NSCLC patients (1). The diagnosis of LM is usually based on the clinical manifestations and a combination of cerebrospinal fluid (CSF) cytology and neuroimaging characteristics. Although CSF cytology remains the gold standard for LM detection, the sensitivity is only 50% (2). Magnetic resonance imaging (MRI) of the brain and spine is also a conventional technique for valuating LM. However, it has been reported that 20-30% of patients with confirmed LM had a false-negative MRI (3, 4). Therefore, it is crucial to identify new approaches to improve the diagnosis and characterization of LM in NSCLC patients. Recent studies have shown that circulating tumor DNA (ctDNA) in CSF can be used to characterize and monitor LM (5), but whether it can function as an early diagnostic biomarker prior to cytological and/or radiographic evidence of LM spread requires further evaluation. In this case, we report the presence of oncogenic mutations in the CSF ctDNA earlier than cytology and MRI-confirmed LM in a lung adenocarcinoma patient and discuss the potential application of CSF ctDNA in the early detection of LM.

## Case report

**Figure 1:**
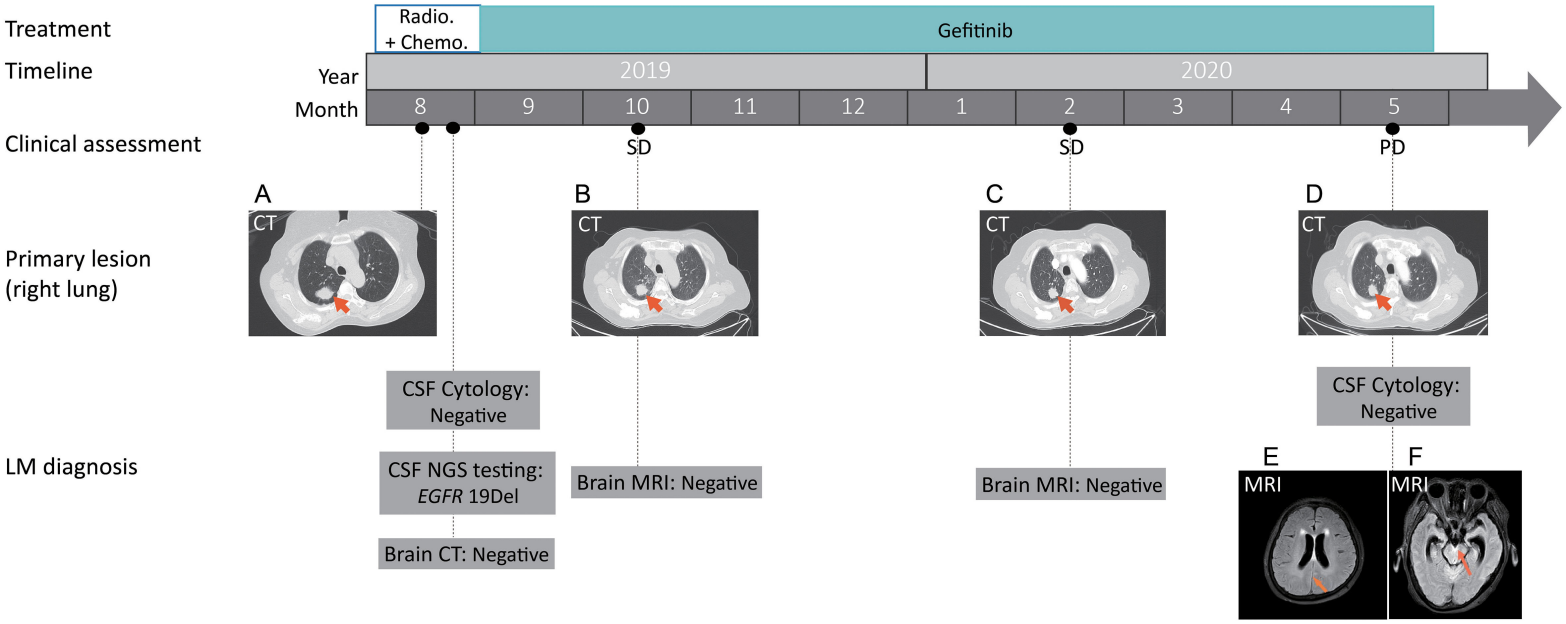
Treatment timeline with representative clinical evaluation and radiologic images. Based on the pathological analysis of the right lung biopsy, together with results from CT scans of the chest, abdomen and radionuclide bone, the patient was diagnosed with a clinical stage IVb LADC, T1cN2M1c according to the eighth edition of The American Joint Committee on Cancer (AJCC) TNM classification.**(A)** Computed tomography (CT) scans showed the primary tumor mass (biopsy site, arrows) in the right lung at the time of diagnosis; **(B, C)** Chest CT showing stable disease (SD) following gefinitib treatment; **(D)** Chest CT showing stable disease in the primary lung site at disease progression. **(E, F)** Magnetic resonance imaging (MRI) showing the potential metastatic site (arrows) in leptomeninges. SD, stable disease; PD, progressive disease; NGS, next-generation sequencing. CSF, cerebrospinal fluid.

A 55-year-old female never-smoker was admitted to the hospital in August 2019 with a pulmonary mass detected by chest computed tomography (CT) during physical examination (**Figure 1A**). Her main complaint was right scapular pain, with a numerical rating scale score of 3. The patient had no other significant pulmonary symptoms, such as cough, tachypnea, or respiratory distress. Further pathological examination of the biopsy of the right lung confirmed lung adenocarcinoma (LADC). Subsequent radionuclide bone scan and abdominal CT revealed multiple bone and liver metastases, respectively. Thus, the patient was diagnosed with stage IVb LADC. To identify potential treatment options, the primary lung tumor biopsy and whole blood normal control were subjected to next-generation sequencing (NGS) tests. While waiting for the test results, the patient was treated with radiotherapy (48Gy/16fractions) to the right scapula targeting the bone metastasis, in combination with one course of chemotherapy (pemetrexed 0.8g d1+carboplatin 0.4g d2). NGS tests revealed a classic epidermal growth factor receptor (*EGFR*) exon 19 (*EGFR* 19Del) in-frame deletion (**Table 1**). Thus, an EGFR-targeted therapy, gefitinib (0.25g qd), was administered. However, ten days following diagnosis, the patient developed severe headache and nausea, which was accompanied by aphasia, restlessness in the limbs, and a sudden vision loss in both eyes, suggestive of brain abnormality. CT scan of the brain showed no obvious abnormality. We also obtained cerebrospinal fluid (CSF) through lumbar puncture for cytological examination and genetic testing to further evaluate brain involvement.

**Table 1:** Genetic alterations identified in the biopsies.

Genes	Alternations	Nucleotide change	Mutant allele frequency	
			Tumor	CSF
*EGFR*	p.L747_P753delinsS	c.2240_2257dellins	53.22%	35.87%
*TP53*	p.R337L	c.1010G>T	40.59%	37.45%
*FLT4*	p.Y361H	c.1081T>C	36.88%	30.18%
*RELA*	p.P384T	c.1150C>A	26.61%	33.53%
*RPTOR*	RPTOR:exon8~BAIAP2:exon14	–	8.33%	8.14%
*MED12*	Intron 25 splice site mutation	c.3577+1G>T	5.97%	8.57%
*ARAF*	p.Y500C	c.1499A>G	1.24%	14.01%
*RARA*	p.A389T	c.1165G>A	–	22.77%
*HGF*	p.G182E	c.545G>A	–	13.06%

-, not detected; CSF, cerebrospinal fluid.

While no heterotypic cells were detected in the CSF, the same *EGFR* 19Del mutation was detected in the CSF ctDNA (**Figure 1**). In both the primary tumor and CSF biopsies, the *EGFR* mutation was detected at a relatively high mutant allele frequency (MAF, 53.22% and 35.87%, respectively) (**Table 1**). Notably, *RARA* c.1165G>A and *HGF* c.545G>A were only present in the CSF, which might be due to inter-tumor heterogeneity between the primary and metastatic lesions (**Table 1**). Despite potential central nervous system (CNS) involvement, the patient did not opt for the more CNS penetrant newer generation EGFR inhibitor, osimertinib, due to financial concerns. With gefitinib treatment, there was no significant change in the sizes of the primary lesion (**Figures 1B, C**) and bone and liver metastasis. Craniocerebral MRIs in October 2019 and February 2020 also showed no obvious abnormalities (**Figure 1**), which indicated stable disease (SD) according to the Response Evaluation Criteria in Solid Tumours (RECIST 1.1) Guideline. Thus, the patient continued on gefitinib treatment. In May 2020, while the primary lung tumor remained stable (**Figure 1D**), brain MRIs showed an abnormal linear signal shadow of the leptomeninges, and clinical evidence of severe headache, vomiting, and aggravated restlessness in the limbs, suggesting the possibility of LM (**Figure 1E, F**). Thus, at the time of disease progression, the patient had derived durable benefit from gefitinib for 9.17 months despite central nervous system involvement early on in the course of the disease, and the patient had only mild adverse reactions during gefitinib administration, including mild itching and fatigue.

## Discussion

Leptomeningeal metastasis is a severe complication in the late stage of malignant tumor, with poor prognosis and limited treatment options. Although positive CSF cytology remains the gold standard for the LM diagnosis, the sensitivity of initial is only 45-50% (3), which can be easily affected by the time and method of specimen collection and detection (6). On the other hand, neuroimaging methods, including CT and MRI, are also valuable in the investigation of LM. MRI demonstrates more sensitivity over CT (7) with a sensitivity of 65% (8). Nevertheless, it has been reported that 20-30% of patients with confirmed LM had a false-negative MRI (3, 4). An accurate diagnosis of LM would rely on both CSF cytology and MRI evaluations, along with clinical manifestations. Regardless, given their low sensitivity, developing other diagnostic approaches is urgently needed.

NGS has emerged as a novel approach for the diagnosis of LM. Recent studies have shown that ctDNA in CSF can be used to characterize and monitor LM. In patients with LM in the BLOOM study, *EGFR*-mutated ctDNA was identified in CSF, the level of which decreased during treatment in correlation with improved neurological function or MRI result (5, 9). Besides, ctDNA analysis can reveal potentially druggable mutations that inform clinical decisions. In our study, the oncogenic mutations identified in the CSF ctDNA were highly concordant with that found in the primary tumor tissue. Although the brain lesion was the eventual cause of progressive disease in this case, the patient remained stable on gefitinib treatment for as long as 9.17 months. We speculate that the patient could have derived more intracranial benefit from drugs such as osimertinib, which was designed for a better CNS penetration to target LM (10).

Furthermore, our case has shown that CSF ctDNA may enable a more sensitive LM diagnosis, which was positive much earlier than any of the CSF cytology or radiographic evidence of LM involvement. In particular, no LM was seen at baseline brain CT, CSF cytology, or the subsequent series of brain MRI tests until the patient experienced PD in May 2020. By contrast, the NGS test of the CSF ctDNA was positive and revealed an oncogenic driver that was consistent with the primary lung tumor tissue from the initial diagnosis. Meanwhile, the patient had been experiencing symptoms such as headache, nausea, aphasia, restless limbs, and a sudden vision loss in both eyes throughout the treatment, which further supports the false negativity of CSF cytology or the radiologic exams. Although we could not confirm whether the patient had LM at the time of disease diagnosis, the patient’s persistent brain abnormality-related symptoms and oncogenic mutations in the CSF strongly support the early involvement of LM. Therefore, our result demonstrates that NGS tests might be able to capture early LM signals in this patient with higher sensitivity than any of the traditional methods. Unfortunately, in this single case, we could not find more direct clues of early LM besides ctDNA results and clinical evidence. Future large-scale studies should be conducted to further verify these findings. Nevertheless, our case suggests combinatorial testing of ctDNA, CSF cytology and MRI might be the trend in medical diagnosis for early detection of disease progression in patients with potential LM involvement.

In general, early diagnosis of LM involvement with sensitive diagnostic methods would be highly valuable in patients with more localized disease at presentation as it provides the patients with more potential surgical and therapeutic options, such as the addition of local brain radiotherapy (LBRT) and the choice of drugs with a better CNS penetration like osimertinib. In particular, for LADC patients with a low level of disease burden, we believe LBRT is a promising approach for controlling brain tumor growth. In our case, although the patient presented with highly metastatic disease at the time of diagnosis, he still could have derived more intracranial benefit, and a longer PFS from osimertinib rather than gefitinib as the eventual cause of progression was an increased size of the brain lesion, if not for financial concerns.

## Data availability statement

The original contributions presented in the study are included in the article/supplementary material. Further inquiries can be directed to the corresponding author.

## Ethics statement

The studies involving human participants were reviewed and approved by the Ethics Committee of Shandong Cancer Hospital and conformed to the provisions of the Declaration of Helsinki (as revised in 2013). Written informed consent was obtained from the patient’s family member for the publication of this case report and any accompanying images.

## Author contributions

SHY conceived and supervised the study. YJB collected clinical data from patients and wrote the paper. QXY, NL and XLL assisted with the clinical data collection and analysis. DW and JWL revised the manuscript and designed the table and image. All authors contributed to the article and approved the submitted version.

## Funding

This work was supported by the Natural Science Foundation of China (NSFC81872475, NSFC82073345).

## Acknowledgments

We would like to thank the patient and his family for giving consent for publication.

## Conflict of interest

Authors JL and DW were employed by Nanjing Geneseeq Technology Inc.

The remaining authors declare that the research was conducted in the absence of any commercial or financial relationships that could be construed as a potential conflict of interest.

## Publisher’s note

All claims expressed in this article are solely those of the authors and do not necessarily represent those of their affiliated organizations, or those of the publisher, the editors and the reviewers. Any product that may be evaluated in this article, or claim that may be made by its manufacturer, is not guaranteed or endorsed by the publisher.
